# Suicide Prevention Audit in Accordance With the NCISH Toolkit – A PAN Trust Audit Across the Four Localities of the Black Country

**DOI:** 10.1192/bjo.2025.10632

**Published:** 2025-06-20

**Authors:** Rhea Mathews, Sharada Abilash, Jaikishan Athi, Tanika Brandaro, Georgia Smith

**Affiliations:** 1 Birmingham Community Healthcare Foundation Trust, Birmingham, United Kingdom; 2 Black Country NHS Foundation Trust, Walsall, United Kingdom; 3 Black Country NHS Foundation Trust, Dudley, United Kingdom; 4 University of Birmingham, Birmingham, United Kingdom

## Abstract

**Aims:** The primary aim of the re-audit was to identify specific areas where key components of the NCISH guidelines were not consistently applied in the care of patients who died by suicide. By addressing these gaps, the Trust seeks to ensure that NCISH-recommended standards are embedded into practice across all patient care pathways. Our objectives were to assess the level of compliance with NCISH standards across all localities where patients died by suicide, evaluate whether the care provided aligns with National Standards and extract key lessons from the cases audited to drive improvements in care delivery and patient safety.

**Methods:** This was a PAN Trust re-audit. A retrospective collection of data was done, and a sample was provided by the trust’s Serious Incident & Inquest Manager. The criteria were any patient known to the trust who completed suicide between April 2022 and March 2023, in line with NCISH guidelines. This collection saw 33 cases, however only 32 cases were audited, as one was withdrawn. This was an increase from the previous 2 years. The cases were split between team members for data collection, and for each case the RiO patient notes and incident reports were used to fill out the audit tool on Microsoft Excel. Data was analysed by the quality improvement team.

**Results:** The audit revealed that no patients died as psychiatric inpatients, but six suicides occurred within three months of discharge. Most were males in their 60s, single, unemployed, and living alone, with depressive illness as the primary diagnosis. Common methods included hanging and self-poisoning, with most suicides occurring at home. Many patients received treatment, though risk assessments and follow-up practices were inconsistent, with structured tools used in only three cases. Lessons learnt emphasize the need for timely, structured post-discharge care, better crisis intervention, enhanced staff training, improved communication between services, and increased access to dual-diagnosis and mental health resources.

**Conclusion:** Post-Review Changes:

Risk assessments now use formal tools, with improved documentation and training on difficult conversations. Collaboration between GPs and mental health services has improved.

Recommendations:

Strengthen post-discharge follow-ups within three months. Provide suicide prevention training for GPs and staff. Increase home monitoring for high-risk patients, especially in Dudley. Launch male-focused mental health campaigns addressing stigma. Enhance inter-service collaboration between GPs, community mental health services, and crisis teams for seamless care coordination. Address systemic resource gaps like staffing and waiting lists.

